# P-1027. Socioeconomic Factors Associated with Adverse Outcomes in Patients with Candidemia

**DOI:** 10.1093/ofid/ofae631.1217

**Published:** 2025-01-29

**Authors:** Hamail Iqbal, Ahmet Dalkilic, Dejan Nikolic, Carlo Foppiano Palacios

**Affiliations:** Cooper Medical School of Rowan University, Summit, New Jersey; Cooper Medical School of Rowan University, Summit, New Jersey; Cooper University Health Care, Camden, New Jersey; Cooper University Health Care, Camden, New Jersey

## Abstract

**Background:**

Candidemia can be a life-threatening disease associated with multiple infectious complications, such as endocarditis and endophthalmitis. With the rise of the opioid epidemic and intravenous drug use, candidemia incidence has increased, particularly among young adult patients. While treatment standards are well-established, data on the impact of socioeconomic factors on patient outcomes is lacking. Here, we aimed to evaluate if candidemia outcomes were associated with various socioeconomic factors.

**Methods:**

A single-center, retrospective chart review was conducted including all patients with candidemia from 2017 to 2022 at an academic medical center. We collected demographic data, culture results, treatment measures, discharge outcomes, and outcomes at 30 and 90 days. Patient census tract codes were used to evaluate socioeconomic markers from the American Community Survey (2020). Descriptive statistics, Chi-square, and Kruskal-Wallis tests were performed.

**Results:**

237 candidemia episodes among 199 patients were included. The mean age was 54 years, 55% were male, 58% were White, and 15% were Latino. Most cases were due to C. albicans (37%), and most patients were treated with micafungin (51%). Patients who did not speak English had a higher hospital mortality (P=0.003), 30-day mortality (P=0.01), and 90-day mortality (P=0.03). Interestingly, patients with a history of substance use disorder (P=0.006) and housing insecurity (P=0.006) were less likely to die inpatient; similar trends were found for 30 and 90-day mortality. Census tract data demonstrated that patients living in areas with more people who do not speak English well were more likely to be readmitted to the hospital within 30 (P=0.02) and 90 (P=0.001) days from discharge

**Conclusion:**

Non-English-speaking patients and those residing in census tracts with higher unemployment had higher mortality. These findings underscore the importance of considering socioeconomic and demographic factors in clinical management and public health strategies aimed at improving outcomes in Candidemia patients. Further research should explore underlying mechanisms driving such disparities.

**Disclosures:**

**All Authors**: No reported disclosures

